# Early evidence of implementation of District Residency Programme: experiences and challenges of residents in Rajasthan, India

**DOI:** 10.1186/s12909-024-05489-w

**Published:** 2024-05-03

**Authors:** Ankit Raj, Shalini Singh, Monika Rathore

**Affiliations:** Department of Community Medicine, Sawai Man Singh Medical College and Hospital, Jaipur, 302004 Rajasthan India

**Keywords:** District Residency Programme, Implementation, Postgraduate Medical Education, National Medical Commission, Residency training

## Abstract

**Background:**

District Residency Programme (DRP) was introduced by National Medical Commission as mandatory three-months training program for postgraduate residents. The program was for the first time implemented in April 2023 in Rajasthan. However, it ran into several teething problems, especially for residents. With a lack of any precedence, this study was planned to explore experiences and challenges of residents posted in DRP.

**Methods:**

Cross-sectional study was conducted at 12 DRP sites attached to SMS Medical College, Jaipur between August-October 2023. A self-administered questionnaire was used to collect information from residents who had completed DRP. Questions were scored on a five-point Likert scale. Mann-Whitney U test and Kruskal-Wallis H test was used to show association.

**Results:**

Only around 17% residents felt that the learning objectives of DRP were fulfilled and nearly 60% residents felt isolated from academic activities and parent department. Over half of the residents were never posted with their concerned specialty services. Around four-fifth residents felt concerned about safety at least sometimes and more than three-fourth residents were dissatisfied with basic amenities. Kruskal-Wallis and Mann-Whitney tests showed significant association of gender and specialisation strata with multiple outcome variables.

**Conclusion:**

The study finds high degree of dissatisfaction among residents towards learning objectives, academic learning, and basic amenities during DRP. There was also a clear lack of specialty-exposure and high concerns of safety, especially for female residents. The study findings should alarm and inform policymakers and administrators to improve DRP implementation so as to better achieve laid objectives.

**Supplementary Information:**

The online version contains supplementary material available at 10.1186/s12909-024-05489-w.

## Introduction

District Residency Programme (DRP) was introduced by the National Medical Commission (NMC) in India as a compulsory three-months residential training programme, part of the course curriculum, for broad specialty postgraduate students admitted from 2021 onwards [1–3]. The objective of the programme was to expose the postgraduate students to District Health System and involve them in health care services being provided at district level. Resident doctors in specialty training would work as members of district teams through “learning while serving” [1]. It would also help strengthen district health system, by filling human resource shortage. District health system in India constitutes of District and Sub-District Hospitals providing services to district- and block-level population respectively. They provide secondary care specialized services in a three-tier health care system and act as referral unit for primary health care services from Primary Health Centres and Sub-Centres.

State governments were tasked with the implementation of DRP [1]. Initially conceived in 2020, the programme was delayed due to COVID-19 pandemic. In 2023, it was first time implemented across India with the state government of Rajasthan implementing the programme in April 2023 [4]. However, residents were posted in DRP at an extremely short notice and without any orientation training. Simultaneously, there were many teething problems, usually associated with any new initiative.

Recent reports have identified multiple problems faced by residents in states across India such as lack of basic amenities (accommodation, food, and sanitation), poor security and safety, and insufficient specialty-focused training [5, 6]. A SWOT analysis of DRP revealed that the major weaknesses of the programme for residents include non-academic work culture in district hospitals, lack of opportunities for professional development, unavailable or poor quality basic amenities, and broken communication with parent department [7]. However, a comprehensive literature review could not identify any study using a scientific approach to assess the implementation of DRP or experience of residents during DRP, especially in the state of Rajasthan. As it was the first year of implementation, there was also a paucity of information on challenges in implementation of DRP.

This study was designed with the primary objective to assess and describe the perception and experiences of postgraduate residents during DRP. Additionally, the study planned to assess and describe the satisfaction with training and challenges faced by postgraduate residents during DRP.

## Materials & methods

A cross-sectional study was conducted among broad-specialty postgraduate residents of Batch 2021 from Sawai Man Singh Medical College, Jaipur, who were also the first batch to complete the mandated three months of DRP. The study was conducted between August-October 2023 at all the 12 DRP sites attached to Sawai Man Singh Medical College, Jaipur, which consisted of nine Sub-District Hospitals and three District Hospitals, across six different districts in Eastern Rajasthan.

Sample size: A minimum sample size of 97 was calculated, at 95% confidence level and 10% absolute error, with an assumption that 50% of the study population would feel that learning objectives of DRP were adequately fulfilled. Simple random sampling was done to select respondents from an available sampling frame of residents posted in DRP. Out of a total of 368 residents who had completed DRP at the time of study, 100 residents (27.2%) were invited to participate in the survey. After checking for completeness and validity of responses, two incomplete questionnaires were excluded from the data analysis.

A pre-tested, self-administered, semi-structured, paper-based, 25-item questionnaire in English was used to collect information from the residents. The questionnaire was developed by the authors using the DRP guidelines, available reports, and feedback from pilot survey on 15 residents. These residents were purposively chosen from Community Medicine, Internal Medicine, General Surgery, and Pathology to represent diversity in experience, and were not included for final study. It was further checked and content-validated by experts. Internal consistency was verified using Cronbach’s Alpha which gave a score of 0.9, indicating excellent reliability. The questionnaire was divided into following sections: baseline demographic information; satisfaction with DRP training; satisfaction with distant academic learning during DRP; satisfaction with basic amenities during DRP; specialty-focused skill training during DRP; and safety and well-being at DRP site. These sections were derived from objectives and guidelines of DRP under Post-Graduate Medical Education Regulations 2023. Questions were scored on a five-point Likert scale on an order ranging between Strongly Disagree (1) to Strongly Agree (5) or Never (1) to Always (5) for positive items and reverse-order for negative items (three questions). Higher scores indicated a more favorable experience of DRP. Two questions were voluntary and open-ended, intending to elaborate on the challenges faced by residents during DRP and to seek recommendations to improve DRP.

The data collected was entered and cleaned on Microsoft Excel version 16.80 and analyzed using SPSS version 28 (SPSS, Inc., Chicago, IL). Ordinal data was represented using proportion and discrete data was represented by median, mode, and range. Mean ranks were generated for each of the ordinal variables. Content analysis was done for two open-ended questions using frequency distribution of common or overlapping terminology. Non-parametric tests (Mann-Whitney U Test and Kruskal-Wallis H Test) were used to show association as the scale was ordinal, variables were non-normally distributed, and frequency of some cells were less than five. *P* < 0.05 was considered statistically significant.

Approval for the study was obtained from the Institutional Ethics Committee at Sawai Man Singh Medical College and informed consent was taken from each participant before seeking response to the questionnaire.

## Results

Of the 98 residents with completed and verified responses, 52% (51) were females and 48% (47) were males. The distribution of responses to question items on five-point Likert scale is summarized in Table 1. The study found that only 17.4% of residents felt that the learning objectives of DRP were fulfilled. Majority of residents did not feel motivated at the end of DRP (67.4%) and felt that they did not receive adequate supportive supervision in DRP (69.6%). Most residents felt that the quality of their postgraduation training suffered during DRP (83.3%) and more than half of residents felt that their parent department did not support their remote participation in academic activities using AV-aids (59.5%).

**Table 1:** Proportion distribution of responses to question items on a five-point Likert scale

	Strongly Agree	Partly Agree	Neutral	Partly Disagree	Strongly Disagree
**Satisfaction with DRP training**					
Felt motivated at the end of DRP	17.39%	6.52%	8.70%	23.91%	43.48%
Satisfied with training received during DRP	10.87%	4.35%	8.70%	19.57%	56.52%
Received adequate supportive supervision	8.70%	4.35%	17.39%	15.22%	54.35%
Training monitored using logbooks & assessment	13.04%	13.04%	17.39%	13.04%	43.48%
Feel learning objectives of DRP was fulfilled	6.52%	10.87%	17.39%	13.04%	52.17%
**Satisfaction with distant academic learning during DRP**					
Quality of post-graduation training suffered	71.43%	11.90%	7.14%	7.14%	2.38%
Parent department provided adequate support	23.81%	9.52%	45.24%	11.90%	9.52%
Parent department used AV-aids to support participation in academic activities	9.52%	9.52%	21.43%	14.29%	45.24%
Received continuous guidance by faculty/guide of parent department	19.05%	16.67%	21.43%	19.05%	23.81%
**Satisfaction with basic amenities during DRP**					
Received decent & safe accommodation	2.44%	7.32%	17.07%	21.95%	51.22%
Had access to clean & hygienic food through mess/canteen	2.44%	4.88%	7.32%	12.20%	73.17%
Had access to clean & safe sanitation facilities	2.44%	7.32%	9.76%	12.20%	68.29%
	**Always**	**Usually**	**Sometimes**	**Rarely**	**Never**
**Specialty-focused skill training during DRP**					
Posted at concerned specialty unit	4.88%	12.20%	19.51%	12.20%	51.22%
Received specialisation exposure	4.88%	2.44%	14.63%	29.27%	48.78%
Learnt specialisation-specific skills	4.88%	2.44%	17.07%	21.95%	53.66%
**Safety and well-being at DRP site**					
Felt concerned about safety	34.88%	27.91%	16.28%	6.98%	13.95%
Experienced work-related stress	13.95%	11.63%	25.58%	25.58%	23.26%
Had access to grievance redressal	11.63%	4.65%	25.58%	27.91%	30.23%

More than half of residents were never posted at their concerned specialty team/unit/section/services (51.2%) and never got to learn specialty-specific skills during DRP (53.7%). 73.2%, 85.4%, and 80.5% residents felt that they did not receive decent & safe accommodation, clean & hygienic food, and clean & safe sanitation facilities respectively. 79.1% residents felt concerned about safety at least sometimes while 58.1% residents felt that they never or rarely had access to grievance redressal (Table 1).

**Fig. 1:**
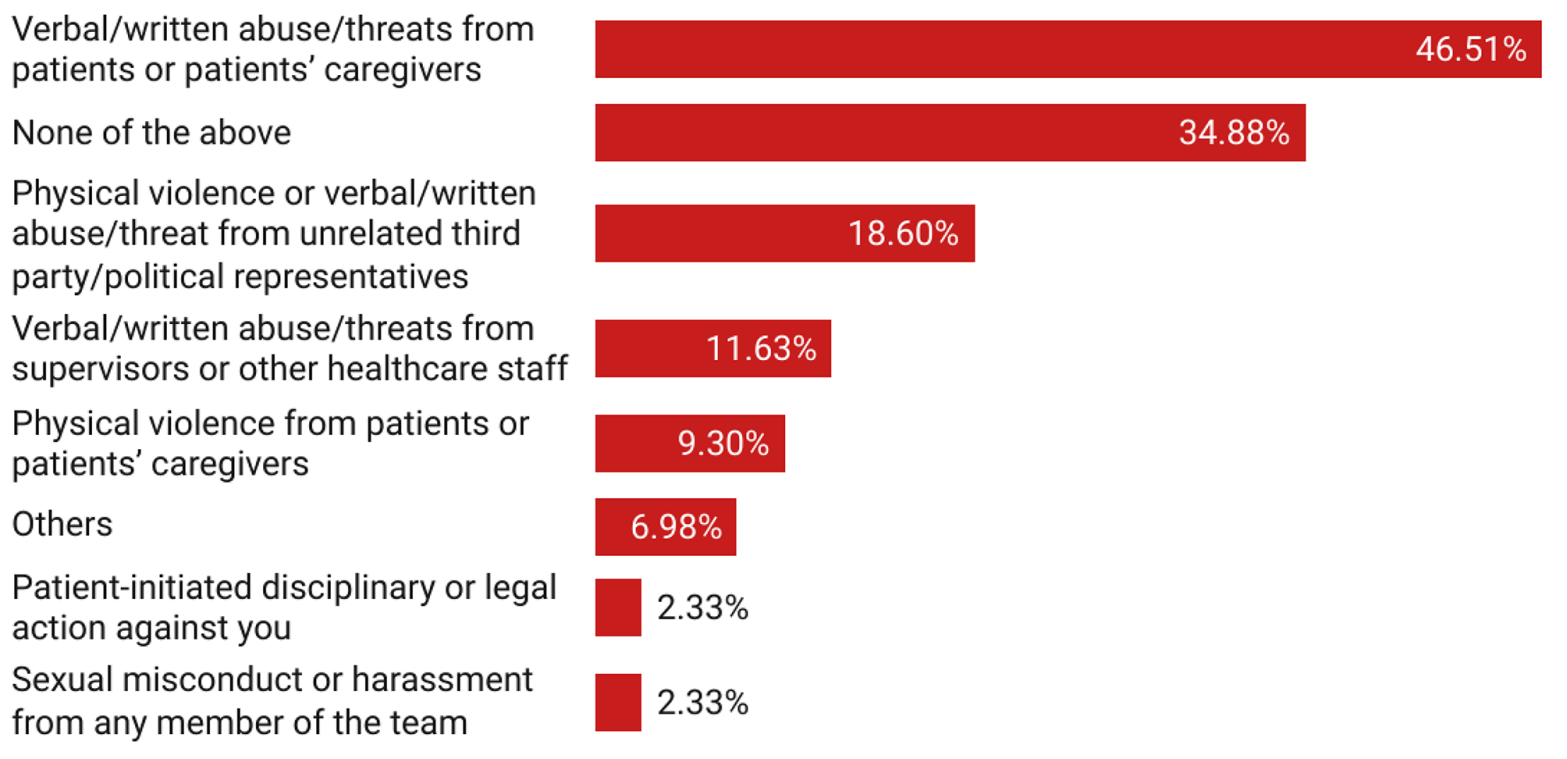
Adverse safety events faced by residents during District Residency Programme

The study showed that the most common adverse safety event faced by residents was “verbal or written abuse/threat from patients or patients’ caregivers” (46.5%), followed by “physical violence or verbal/written abuse/threat from unrelated third party/political representatives” (18.6%). 34.9% of the residents did not face any adverse safety event while 6.9% residents faced other issues such as “pressure to write unnecessary investigations” (Fig. 1).

Statistical analysis using Mann-Whitney U test showed significant difference in mean ranks between male and female residents for three different ordinal variables (Table 2). Female residents rated being significantly less satisfied with supportive supervision and sanitation facilities than male residents, and more likely to be concerned about safety than male residents (*p* < 0.05). No significant association was noted between gender and other variables in the study.

**Table 2:** Mann-Whitney U Test showing significant association between gender and three dependent variables (*p* < 0.05)

	Mean Rank (Male)	Mean Rank (Female)	*p*-value
Received adequate supportive supervision	2.22	1.74	0.04
Had access to clean & safe sanitation facilities	1.90	1.38	0.02
Felt concerned about safety	2.71	2.05	0.04

**Fig. 2:**
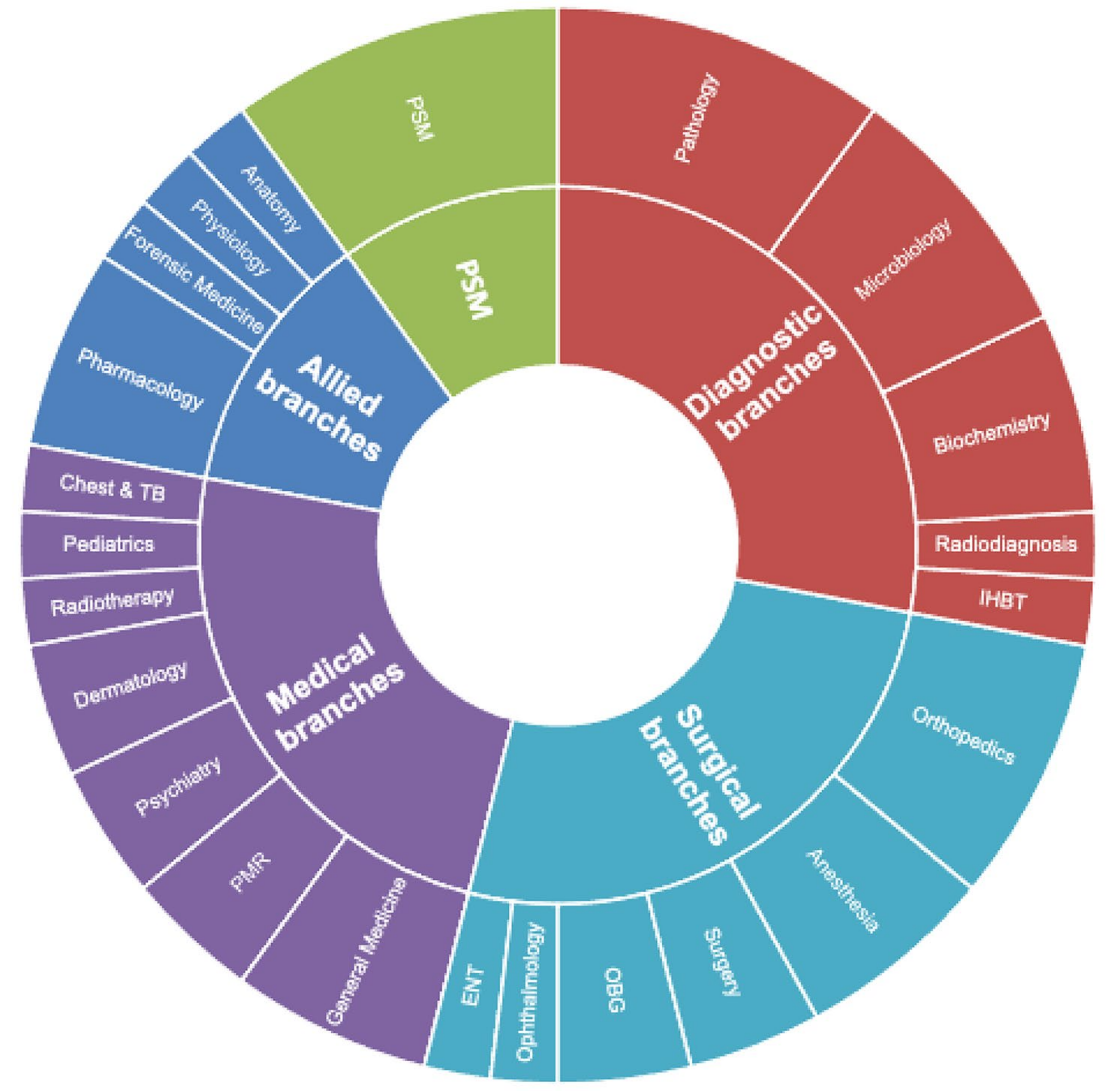
A sunburst diagram showing specialization distribution of residents divided into five groups based on professional homogeneity and relevance to DRP.

For sub-group analysis, specialization distribution of residents was divided into five groups based on academic and professional homogeneity and relevance to objectives of DRP. Maximum respondents were from diagnostic branches (28.5%), followed by surgical branches (26.5%), medical branches (24.5%), allied branches (12.3%), and Community Medicine/Preventive & Social Medicine (10.2%) (Fig. 2). Community Medicine/Preventive & Social Medicine was kept as a separate group as learning outcomes of this branch highly correlates to DRP objectives and these residents stand to gain most from exposure to district health system.

Kruskal-Wallis H and post-hoc test was performed to check for statistically significant association and pairwise comparison respectively between specialty groups. Table 3 shows significant difference in mean ranks between 16 pairs across eight dependent ordinal variables (*p* < 0.05). Medical branches rated feeling significantly less motivated than allied or diagnostic branches, were less satisfied with DRP training than allied branches, and learnt specialty-specific skills less often than allied branches. Residents of community medicine rated being most dissatisfied by support from parent department than all other specialty groups. Surgical branches residents rated feeling significantly higher satisfied with accommodation and food than community medicine, diagnostic, or medical branches.

**Table 3:** Kruskal-Wallis H Test showing significant association between specialization groups and dependent variables (*p* < 0.05)

	Mean Ranks					*p*-value	Post-hoc pairwise comparison (significant difference)
	Allied branches	PSM	Diagnostic branches	Medical branches	Surgical branches		
Felt motivated at the end of DRP	3.6	2	3	1.4	2	< 0.001	Allied/Diagnostic branches v/s Medical branches
Satisfied with training received in DRP	2.6	1.57	2.42	1.3	1.92	0.049	Allied branches v/s medical branches
Training monitored using logbooks & assessment	2.8	1.43	3	2.2	2.33	0.041	PSM v/s Diagnostic branches
Had access to grievance redressal	2.2	1.71	2.91	2.2	2.6	0.02	PSM v/s Diagnostic branches
Parent department provided adequate support	4.2	1.71	3.45	3.5	3.44	0.001	PSM v/s Allied/Diagnostic/Medical/Surgical branches
Learnt specialisation-specific skills	2.7	1.43	2.1	1.4	1.89	0.033	Medical branches v/s Allied branches
Received decent & safe accommodation	2.2	1.57	1.6	1.5	2.67	0.009	Surgical branches v/s PSM/Diagnostic/Medical branches
Had access to clean & hygienic food through mess/canteen	2	1.29	1.3	1.2	2	0.015	Surgical branches v/s PSM/Diagnostic/Medical branches

One month was the median (range: 1–3 months) and most commonly (58%) recommended duration of DRP as sufficient by the residents, as compared to current prescribed norms of three months.

Content analysis was performed for two open-ended questions. When asked to elaborate or add challenges faced by residents during DRP not covered in the questionnaire, many expressed unavailability of transportation facilities (14.3%) and lack of any orientation training before beginning DRP (11.2%). Few residents also expressed that service delivery and training during DRP often went unsupervised, with their supervisors (Medical Officers) from DRP sites often absent or only briefly present (6.1%). One resident also shared about difficult and authoritative behaviour of District Residency Programme Coordinator which affected their mental well-being.

When asked for recommendations through an open-ended question, many residents wished for the District Residency Programme to be withdrawn or reduced in duration (32.1%). Most common recommendations were to improve accommodation facilities (24.5%) and provision of specialty-focused training (12.3%). A few recommended to shift the DRP to third year of residency, from current second year (3.1%).

## Discussions

DRP was launched with the objective to expose postgraduate trainee doctors to District Health System and for them to learn to provide services close to community (learning while serving) [1, 2]. However, our study finds that nearly two-third of residents who completed DRP feel that DRP training failed to fulfill its learning objectives and around three-fourth were not satisfied with training received during DRP. Some degree of dissatisfaction is expected in initial stages of any new initiative, but our study finds a worrying level of overwhelming dissatisfaction with DRP training. Additionally, medical branches rated being dissatisfied with training more than any other branch and significantly more than allied branches. Medical branches also felt least motivated and allied branches most motivated at the end of DRP. These findings are contradictory to intuitive notion that DRP would satisfy learning outcomes and hence motivate medical branches more than allied branches due to more unrestricted clinical exposure.

NMC mandates that the quality of training during DRP shall be monitored by logbooks, supportive supervision, and continuous assessment of performance [1, 3]. Yet findings from our study reveal that more than two-third residents did not receive satisfactory supportive supervision. Additionally, male residents were more likely to receive satisfactory supportive supervision than female residents. This could be because peripheral district facilities are more prone to non-academic work environment and male-dominated hierarchy than medical colleges.

NMC encourages continued and remote participation of residents in academic activities of parent department/medical college [1]. A SWOT analysis had identified broken communication from parent department during DRP as one of the major weaknesses [7]. This study found that majority of residents feel that they are isolated from academics and parent department, with a lack of provision for remote academic participation. Over half of the residents felt that their parent department did not use AV-aids sufficiently to support their remote participation in academic activities. Community Medicine residents particularly ranked their satisfaction of support from parent department significantly less than any other branch.

The goal of postgraduate medical education is to produce competent specialists and at the end of the training the graduate should demonstrate sufficient skills and knowledge of the concerned specialty [1, 2]. NMC endorsed that residents should work as specialty doctors with district teams during DRP and that they be posted with the concerned/aligned specialty team/services at the District Health System while serving in areas pertaining to their specialty [1, 3]. However, this study reveals that there was a lack of specialty-focused work exposure and skills-learning among residents in DRP. Over half of the residents were never posted with the concerned/aligned specialty team/services while over three-fourth never got to learn specialty-specific skills. The findings reaffirm a report from Tamil Nadu that identified resident doctors’ opposition to DRP due to a lack of exposure to parent specialty [6]. This is a major concern as residents are missing out on vital exposure to their specialty for three months in a three year learning period.

Safety and security are major issues for well-being of postgraduate trainees as workplace violence against doctors has risen in recent past in India [8]. Our study revealed some serious concerns related to safety and well-being of resident doctors working in district health system under DRP. Nearly four out of five residents felt concerned about their safety at least sometimes during DRP and over half of the residents faced some kind of safety issue. Nearly half of the individuals had experienced verbal or written abuse/threat from patients or patients’ caregivers. Many residents had also faced physical violence at the workplace while a few even reported sexual misconduct or harassment from team members. Female residents were more likely to feel concerned about safety than males. These findings corroborate reports from Maharashtra, Andhra Pradesh, Telangana, and Madhya Pradesh that identified poor safety and security faced by residents during DRP [5]. These safety issues are especially concerning as DRP is a residential training away from the safe confines of medical colleges in an unfamiliar environment devoid of support. Any adverse safety issue severely demoralizes and disincentivizes residents from providing services closer to community in district health system.

Access to basic amenities at workplace is one of the basic human rights and key to decent working conditions. However, this study found concerning elements of lack or ignorance of basic amenities for residents during DRP. More than three-fourth of residents felt they did not have satisfactory access to decent and safe accommodation, clean and hygienic food, and clean and safe sanitation facilities. Female residents especially faced concerns regarding clean and safe sanitation facilities. A report from multiple states identified lack of access to basic amenities as a noteworthy challenge faced by residents during DRP [5]. NMC has assigned state governments with the responsibility to provide appropriate amenities including suitable accommodation and security, especially for female residents [3]. Stakeholders implementing the program at state level should immediately seek corrective measures on basic amenities and security, to avoid untoward incidents and demoralization of residents. Over half of residents also opined reduction in DRP duration to one month from the current mandated norm of three months.

A major strength of this study is the novelty as 2023 was the first year of implementation of DRP and this study was conducted on one of the first batch of residents undergoing DRP. To authors’ knowledge and a comprehensive literature review, this is the first study to document and quantify the experience of residents with DRP. Findings from this study will inform policymakers and administrators in optimizing the implementation of DRP so that residents meet the learning objectives while feeling safe and comfortable. The study had all the limitations of a cross-sectional survey. As the study population was from a single medical college, it may not be representative of the experience of residents from other medical colleges or other states. Large-scale, national study should be conducted, preferably with a qualitative component, to better understand the experience of residents undergoing DRP.

## Conclusion

This study is one of the earliest pieces of evidence of implementation of DRP in India that documents the experience of programme by resident doctors. Our study reports a high degree of dissatisfaction among postgraduate residents regarding learning objectives and academic activities during DRP. Residents experience a lack of specialty-exposure and disconnect from their parent department during the course of DRP. There are also pronounced concerns regarding basic amenities, personal safety, and security, especially for female residents. Findings from this study should inform medical educators, policymakers, and administrators to improve the implementation of DRP and enhance experience of residents in learning while serving.

### Electronic supplementary material

Below is the link to the electronic supplementary material.

Supplementary Material 1

## Data Availability

Data is provided as a supplementary information file.
